# Assessing green manure impact on wheat productivity through Bayesian analysis of yield monitor data

**DOI:** 10.3389/fpls.2024.1323124

**Published:** 2024-03-27

**Authors:** Niko Gamulin, Miroslav Zorić, Đura Karagić, Sreten Terzić

**Affiliations:** Agro R&D, Login EKO d.o.o., Aradac, Serbia

**Keywords:** green manure, soil fertility, Bayesian analysis, telemetry data, MCMC

## Abstract

Agronomy research traditionally relies on small, controlled trial plots, which may not accurately represent the complexities and variabilities found in larger, real-world settings. To address this gap, we introduce a Bayesian methodology for the analysis of yield monitor data, systematically collected across extensive agricultural landscapes during the 2020/21 and 2021/22 growing seasons. Utilizing advanced yield monitoring equipment, our method provides a detailed examination of the effects of green manure on wheat yields in a real-world context. The results from this comprehensive analysis reveal significant insights into the impact of green manure application on wheat production, demonstrating enhanced yield outcomes across varied landscapes. This evidence suggests that the Bayesian approach to analyzing yield monitor data can offer more precise and contextually relevant information than traditional experimental designs. This research underscores the value of integrating large-scale data analysis techniques in agronomy, moving beyond small-scale trials to offer a broader, more accurate perspective on agricultural practices. The adoption of such methodologies promises to refine farming strategies and policies, ultimately leading to more effective and sustainable agricultural outcomes. The inclusion of a Python script in the appendix illustrates our analytical process, providing a tangible resource for replicating and extending this research within the agronomic community.

## 1 Introduction

Green manure is the practice of incorporating plants into the soil as a nutrient source, has gained increasing attention in recent years due to its potential to enhance soil fertility, improve crop productivity, and promote sustainable agricultural practices Ye et al. (2014); Cai et al. (2019); Ma et al. (2021). Additionally, the escalating expenses linked with chemical fertilizers have underscored the significance of exploring and implementing green manure practices Tanveer et al. (2019); Li et al. (2020); Lei et al. (2022). Green manure offers ecological services by harnessing the power of natural processes to protection against soil erosion, reduction of nutrient losses, improvement of soil and water quality, and to some extent, the reduction of occurrence of pests and weeds Dabney et al. (2001); Hartwig and Ammon (2002); Ryder and Fares (2008); Dorn et al. (2015). Crops such as leguminous species have been used widely as green manures to increase the available nitrogen in the soil and organic matter in general Vyn et al. (2000); Thorup-Kristensen et al. (2003).

The positive impact of field peas as green manure crops on wheat yield and quality is well-established. Wheat stands as a pivotal crop in human civilization, being the most extensively cultivated cereal globally, with over 220 million hectares planted annually across various climatic zones on all continents. Therefore, even a modest enhancement in wheat production sustainability, such as through greenhouse gas emission reduction, can exert a significant impact on the global environment. However, the effects of legume crops on subsequent cereals are highly variable Kirkegaard et al. (2008); Preissel et al. (2015) and pose challenges in predictability. This variability can be attributed to diverse environmental conditions and agronomic practices.

Agricultural researchers traditionally conducted small-scale field trials using appropriate experimental designs in order to evaluate or compare the performance of different treatments, crop varieties, or management practices. Classical experimental designs are based on Fisher’s principles: randomization, replication and local control Fisher et al. (1960). Statistical analysis employed by appropriate linear model approaches is based on the key assumption that the model errors are independent, identically distributed and with constant variance Piepho et al. (2013). Frequently, all these assumptions are violated due to omnipresent within-field spatial variability. It implying the presence of the small scale variability or among plot correlation. Gilmour et al. (1997) divided the experimental design variation into three meaningful types of variation i.e., (i) local trend which reflect small variations in soil fertility and moisture; (ii) large scale variation reflects the global trend of variation typically along the row or column directions and (iii) extraneous variation caused from agricultural management practices that may have a recurrent pattern (for example, direction of planting or harvesting). Availability of the linear mixed models lead the development of the powerful methodologies and approaches for analysis of data from well-designed randomized experiments Besag and Kempton (1986); Grondona et al. (1996) which gained in precision and power of the conclusions Marchant et al. (2019).

In contrast to small-scale field trials, the large-scale field or on-farm trials are more variable and less precise but more representative when compared with a standard agronomic practice in a given production region Piepho et al. (2011). Intensive developments in the field of precision agriculture open the possibilities for conducting of the on-farm experiments to compare the different agronomic practices or to test the conclusions from a small-scale field experiments with advanced possibilities in data collection with a spatial resolution such as the yield monitor data throughout the standard machineries Paccioretti et al. (2021); Hegedus et al. (2023).

Previously mentioned techniques for analysis small-scale experiments cannot be readily applied for data analysis of the on-farm experiments due to their complexity, heterogeneity and spatial scale variability. There are large number of methodological approaches for the analysis of on-farm experiments ranging from geospatial regression models to Bayesian statistical methods Kyveryga (2019); Cho et al. (2021); Paccioretti et al. (2021); Hegedus et al. (2023).

Bayesian statistics is a “yet another” and powerful framework for making informed decisions from the data Kruschke (2015). It allows researchers to incorporate existing knowledge, beliefs, or previous data into the model. For example, existing *a priori* knowledge about physiological parameters of the crop, soil properties, as well as weather variables can be used for the improvement of the prediction accuracy. Furthermore, Bayesian statistics provides a framework for explicit modeling uncertainty of various sources such as weather conditions or occurrence of pests and plant diseases Bi and Chen (2011).

Bayesian statistics played a crucial role in our proposed model for estimation of the green manure effect on the commercial crop. Our approach, which involves updating the probability for a hypothesis as more evidence or information becomes available, allowed us to incorporate both the inherent variability in data and the uncertainty in our prior beliefs into Bayesian model. This provided a distinct and robust understanding of the effectiveness of green manure, and allowed us to make more informed recommendations for its use on production fields.

Green manure is an important natural and sustainable tool for maintaining the production of the healthier food while reducing the negative environmental impact of agriculture on living environment and making agriculture more resilient on climate change. To our best knowledge this is first large-scale study to compare the effectiveness of the green manure on yield of commercial crop using Bayesian estimation model and spatial resolution of yield monitor data. The primary objective of this study is to demonstrate the proposed model functionality using three real field datasets from two growing seasons. Secondly, the theoretical behind of our proposed Bayesian model will be outlined. The Python script for the easy implementation of the proposed model will be described.

## 2 Materials and methods

### 2.1 Site description

**Figure 1:**
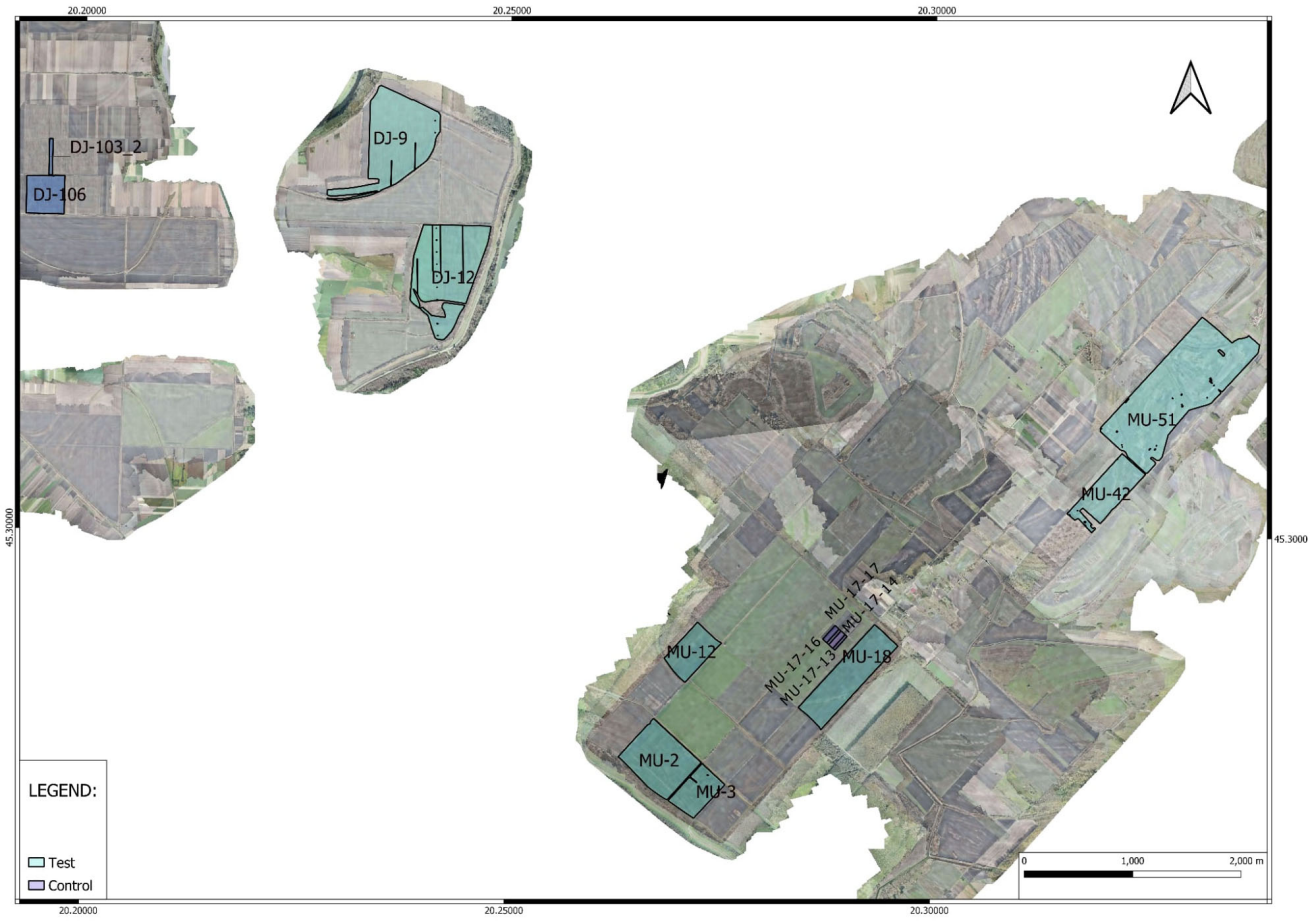
Aerial view of agricultural fields in Mužlja and Ðurđevo Vojvodina, Serbia, where data was analyzed for the year 2022. The legend differentiates between fields treated with green manure (Test) and untreated fields (Control), providing a visual representation of the experimental design for the assessment of green manure’s impact on crop yield.

**Figure 2:**
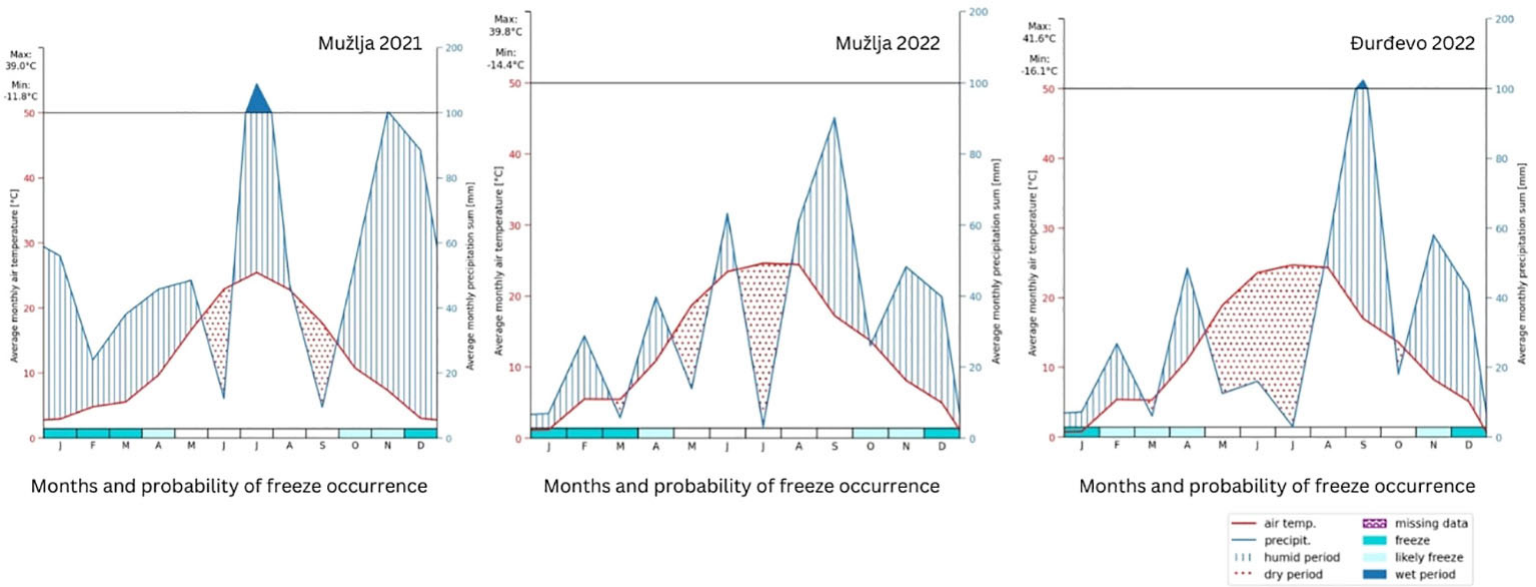
Comparison of seasonal weather data for MU (2021 and 2022) and DJ (2022), illustrating average monthly air temperatures, precipitation sums, and freeze probabilities. The x-axis categorizes months alongside freeze probabilities, while dual y-axes display temperatures and precipitation, respectively.

The sites (**Figure 1**) climatic zone is characterized by a long-term annual average temperature of 11.1°C and a frost-free period extending over 180 days. The region typically receives an annual precipitation sum of 580 mm. However, the year 2021 experienced precipitation levels exceeding this multi-year average by 88 mm. Despite this overall increase, a critical drought period lasting from late May through June significantly affected the critical growth stages of wheat, namely flowering and grain filling, due to reduced nutrient availability and constrained yields. This drought was exacerbated by an increase in the annual air temperature by 1.4°C above the long-term average, further stressing the crops. In contrast, 2022 saw a reduction in precipitation, with the Mužlja (MU) and Ðurdevo¯ (DJ) locations receiving 153 mm and 175 mm less than the multi-year average, respectively. Drought periods were notably severe, especially from May to June and June-July for MU, and from May through the first decade of August for DJ. The annual temperature also increased by 2.1°C, affecting both soil moisture and air humidity (**Figure 2**).

The soil across the experimental sites is classified as Pellic Vertisol (Aric, Mollic, Gleyic, Raptic) (VR-pe-ai.mo.gl.rp) according to the WRB classification. This heavy clay soil, with a combined silt and clay content ranging from 76-85%, exhibits significant physical characteristics that influence its agricultural potential. Detailed information on the soil texture is provided in **Table 1**. The soil’s structurelessness when wet and its propensity to crack deeply (up to 50 cm) when dry, alongside high bulk density, substantial total porosity, and very low water infiltration rates, underscore a prevalence of micropores. These features contribute to its high water holding capacity, as detailed in **Table 2**, yet hinder drainage and soil aeration. During spring, the soil remains cool and moist for an extended period, effectively shortening the vegetation season.

**Table 1:** Soil texture (%) up to 30 cm in depth.

Location	Coarse sand (2-0.2 mm)	Fine sand (0.2-0.02 mm)	Silt (0.02-0.002 mm)	Clay (<0.002 mm)
MU	0.60	13.90	24.07	61.44
DJ	1.09	22.43	26.68	49.80

**Table 2:** Physical attributes of soil up to 30 cm in depth.

Location	Physical Properties				Water retention (% weight)		
	Specific weight (g cm^-3^)	Bulk density (g cm^-3^)	Porosity total (vol. %)	Filtration (K-Darcy (cm s^-1^)	33 kPa	625 kPa	1500 kPa
MU	2.38	1.55	30.16	5.43x10^-4^	47.24	40.34	35.61
DJ	2.52	1.38	35.13	9.16x10^-4^	45.55	41.67	28.82

Acidic in nature, the soils at both locations have a very low calcium carbonate (CaCO3) content and a moderate to high level of organic matter, contributing to their fertility profile. Available phosphorus levels are very low, while potassium levels are adequate (**Table 3**). It is important to note that the last application of synthetic mineral fertilizers occurred in the autumn of 2018, as part of the transition to organic farming practices, ensuring that residual effects on soil nutrient status during our study period were minimized.

**Table 3:** Chemical attributes of soil up to 30 cm in depth.

Location	pH in 1M KCl	ECe 25°C dS/m	CaCO_3_ (%)	SOC (%)	Total N (%)	AL-P_2_O_5_ (mg/100g)	AL-K_2_O (mg/100g)
MU	5.05	0.479	0.11	4.07	0.261	4.74	33.86
DJ	5.71	0.502	0.24	3.38	0.17	3.04	23.19

#### 2.1.1 Plant material description

##### 2.1.1.1 Green manure crop

Field peas (Pisum sativum var. sativum), the local cultivar NS Mraz, were utilized as a green manure crop. This cultivar is a winter semidwarf field pea variety, characterized by the development of lush vegetative biomass reaching an average height of 75-80 cm at the flowering stage (BBCH-scale 65). It is an early-maturing variety, with relatively thick and sturdy stems, exhibiting good standing ability and favorable tolerance to major pea diseases. Additionally, it demonstrates excellent tolerance to low temperatures, even in the absence of snow cover. The achieved yields of green biomass, dry matter, content of key macroelements, and ash are presented in **Table 4**

**Table 4:** Winter field peas, cv. NS Mraz, aboveground biomass yield and chemical composition at flowering stage (BBCH-scale 65).

Location	Fresh matter yield (kg m^−2^)	Dry matter yield (kg m^−2^)	Macronutrients content (%)				Ash content (%)
			N	P	K	C	
MU21	2.20	0.431	3.867	0.29	2.081	41.74	9.64
MU22	2.48	0.610	3.58	0.43	2.69	39.04	9.79
DJ22	2.83	0.481	3.977	0.334	2.014	42.01	9.27

##### 2.1.1.2 Wheat crop

The French winter soft wheat (Triticum aestivum L.) variety Solenzara has been utilized across all environments. It is recognized for its notable yield potential and good disease tolerance. It demonstrates satisfactory performance even in heavy clay soils and semi-arid climates, making it a viable option for various agricultural conditions, including organic farming systems.

### 2.2 Data

Our study meticulously curated yield monitoring data from agricultural fields for a detailed analysis of crop yields influenced by green manure application. The initial phase in 2021 concentrated on fields within MU, where we established two sets of fields: test fields (TF) which had green manure applied, and control fields (CF) which did not, to set a benchmark for yield comparison within this locale. For the 2022 season, we extended our observation to include a different set of fields in MU, alongside newly incorporated fields from DJ, maintaining the division into TF and CF in both areas for our comparative study. These fields were selected based on their historical management sequences being consistent across both TF and CF, ensuring comparability with the sole variable being the application of green manure. This selection process, in conjunction with aerial imagery presented in the article, allowed for a detailed bifurcated analysis of two geographically proximate locations—separated by roughly six kilometers—thereby enhancing the robustness of our comparative framework. The dataset’s comprehensive details, such as the number of measurements, field counts, and total area sizes for each location, are systematically listed in **Table 5**.

**Table 5:** Summary of dataset characteristics per location.

Season	Location	Number of fields	Abbrev.	Total area (ha)	Total number of meas.	Sample size
2021	MU	21	MU21	190	15847	Test: 1104, Ctrl.: 644
2022	MU	13	MU22	110	80137	Test: 964, Ctrl.: 964
2022	DJ	4	DJ22	110	40959	Test: 423, Ctrl.: 188

In this table, “Abbrev.” stands for abbreviation, “Meas.” stands for Measurements, AND “Ctrl.” stands for Control.

**Figure 3:**
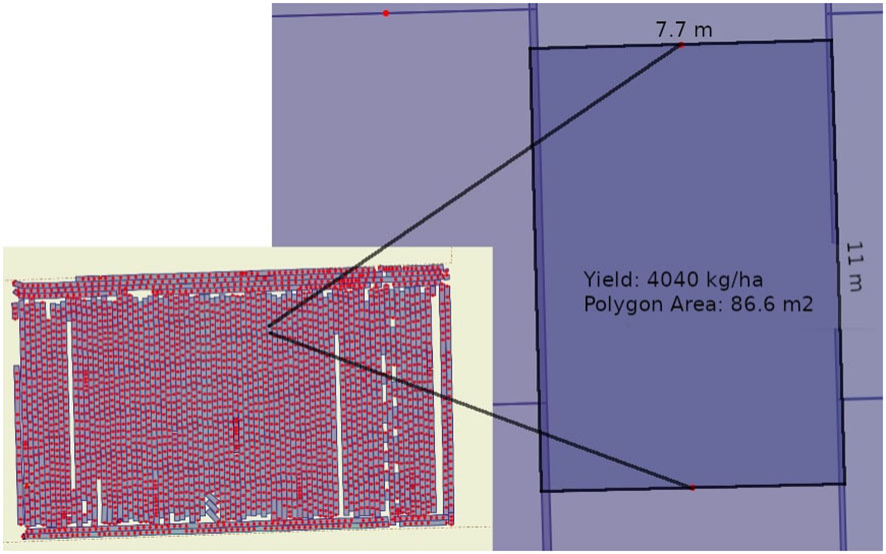
This image showcases the meticulous segmentation of agricultural fields into discrete polygons, illustrated by a detailed example with a yield of 4040 kg/ha over an 86.6 m^2^ area. Such precise partitioning is vital for capturing the subtle variability across the field, allowing for a nuanced analysis of crop yield determinants.

To further refine our experimental design, as depicted in **Figure 3**, the fields were segmented into precisely measured polygons to serve as distinct observational units. These polygons were dimensioned to align with the width of the harvesting equipment and the length determined by the interval of measurement combined with the harvester’s speed. This granular method of field division was critical in capturing the subtle variances within each field. Through this structured approach, we ensured that the fields within each pair—TF and CF—were not only similar in terms of past management practices and soil types, with slight variations between MU and DJ, but also that they were geographically aligned to minimize environmental variability. Thus, our experimental design allowed for a nuanced analysis that could accurately evaluate the impact of green manure on crop yield variations, controlling for other potential confounding factors.

#### 2.2.1 Data preparation

##### 2.2.1.1 Filtering

Our data filtering process aimed to bolster the reliability and validity of the subsequent analysis. Initially, outliers were rigorously identified by applying a statistical threshold based on the 3-sigma rule. This rule posits that in a normal distribution, nearly all values (99.7%) lie within three standard deviations (sigma) of the mean. Measurements falling outside this range were deemed outliers and excluded from the datasets. This criterion was chosen as it effectively removes extreme deviations that could disproportionately influence the analysis, ensuring a focus on data that accurately represents the central tendency and variability of our sample populations.

##### 2.2.1.2 Sampling

In MU22 and DJ22 datasets, we encountered a significant disparity in area sizes between TF and CF, which necessitated the implementation of a sampling process to equilibrate the representation of each field within our analysis. For MU22, we had 423 measurements for the TF and 188 for the CF, while for DJ22, both the TF and CF contained 964 measurements each.

To address this, we employed a stratified random sampling technique. Each field, whether a test or control, was treated as a stratum from which we randomly selected a proportional number of measurements. This approach ensured that each field contributed equally to the final analysis, irrespective of its size, thus maintaining the integrity of the comparison between the TF and CF groups. For MU22, this meant random sampling from the TF and CF measurements to balance the two groups. In contrast, for DJ22, since the number of measurements was already balanced, we ensured that the selection was random and representative.

This sampling strategy served three key purposes:

1. It guaranteed that each field, regardless of acreage, had an equivalent influence on the overall results, thereby preventing larger fields from unduly affecting the analytical outcome.
2. It reduced the potential for bias that might favor the test group if the TF areas were significantly larger, as larger sample sizes can lead to overestimation of effects.
3. It increased the comparability between the TF and CF groups, thereby enhancing the reliability and validity of the study’s conclusions.

In summary, this meticulous filtering and sampling process was designed to refine our datasets, ensuring that they accurately reflect the true effects of green manure application on crop yield without distortion from outliers or uneven field representation.

### 2.3 Theoretical framework

#### 2.3.1 Bayesian analysis and Markov Chain Monte Carlo techniques

In our study, we employed Bayesian analysis, a statistical approach that combines prior knowledge with observed data to update the probability of a hypothesis Kruschke (2015); West (2016). This method is particularly advantageous when dealing with complex systems or limited data, as it facilitates a more comprehensive interpretation of results Kruschke (2010).

We also utilized Markov Chain Monte Carlo (MCMC) techniques, a class of algorithms designed to approximate complex probability distributions often encountered in Bayesian analysis Besag et al. (1995). MCMC methods generate random samples from a target distribution by simulating a Markov chain, a sequence of random variables where each variable depends solely on its immediate predecessor Karras et al. (2022). Over time, the chain converges to the desired distribution, enabling the estimation of various quantities of interest Robert (1995).

In the context of evaluating the effectiveness of green manure, these statistical techniques were instrumental in analyzing the extensive yield monitor data. Traditional agronomic research often relies on comparing control and test groups comprising microplots, which may not adequately account for field heterogeneity or other factors influencing crop yields.

By harnessing the capabilities of Bayesian analysis and MCMC techniques, our study surmounted these limitations. The extensive yield monitor measurements offered a more detailed and accurate representation of each field, facilitating a deeper understanding of the relationships between green manure application and crop yields. This approach allowed us to account for field heterogeneity and other confounding factors, yielding more robust and statistically significant results.

##### 2.3.1.1 Estimating priors

In this study, the posterior distribution is derived from the dry yield measurements observed in both scenarios - with and without the application of green manure. Each measurement is modeled as a function of the mean (*µ*) and standard deviation (*σ*). These parameters, *µ* and *σ*, are priors that are computed given the posterior, under the assumption that the distribution follows a Normal (Gaussian) pattern. The direct analytical calculation of prior values *µ* and *σ* can be complex. Therefore, it is standard practice to estimate these values using MCMC methods, which provide a powerful and efficient approach for approximating these parameters.

MCMC methods are a class of algorithms for sampling from a probability distribution Van Ravenzwaaij et al. (2018). They construct a Markov chain that has the desired distribution as its equilibrium distribution. The states of the chain, after a large number of steps, are then used as samples from the desired distribution.

The MCMC method operates in the following manner:

1. Initialization: Start from any position. This could be a random position or an educated guess.
2. Iteration: For each iteration of the algorithm, propose a new position. The method to propose new positions is specific to the MCMC algorithm being used.
3. Acceptance or rejection: Based on the likelihood of the new position (which is calculated from the desired distribution), decide whether to move to the new position or stay at the current position. This decision is made using the acceptance rule, which, in the case of the Metropolis-Hastings algorithm, for example, accepts movements that increase the likelihood and also sometimes accepts movements that decrease it.

This process is repeated many times. The positions form a Markov chain, where each position is dependent only on the previous one, and after a large number of iterations, the distribution of positions will approximate the desired distribution.

In the context of Bayesian inference, MCMC is used to sample from the posterior distribution of the parameters. In the provided code, the No-U-Turn Sampler (NUTS) Hoffman et al. (2014), an extension of the Hamiltonian Monte Carlo, is used for this purpose. The foundation of MCMC technique is anchored in Bayes’ theorem Harney (2003), as presented in Equation (1).


(1) $$P(\theta |D)=\frac{P(D|\theta )P\left(\theta \right)}{P\left(D\right)}$$

The components of Equation (1) can be delineated as:

- *P*(*θ*|*D*) is the posterior distribution of the parameters (*θ*) given the data (*D*). This is what we want to compute.
- *P*(*D*|*θ*) is the likelihood of the data given the parameters.
- *P*(*θ*) is the prior distribution of the parameters.
- *P*(*D*) is the probability of the data, also known as the evidence.

MCMC methods, including NUTS, make it possible to sample from the posterior distribution without needing to compute the evidence P(D), which can be computationally expensive or even intractable.

By employing MCMC on the dry yield measurements, we can derive estimates for the most probable values of *µ* and *σ*. In essence, the absolute discrepancy between the reconstructed dry yield values and the observed measurements should be minimized. This process allows us to achieve an optimal estimation of the parameters, thereby providing a robust model for the underlying dry yield distribution.

##### 2.3.1.2 Calculating contrast distribution

Upon determining the statistical properties of the yield measurements distribution for both scenarios-with and without the application of green manure - simulations were conducted to reconstruct the statistics for both cases. The effectiveness of green manure was evaluated by comparing the mean values of posterior distributions, achieved by calculating the contrast distribution.

The process of calculating the contrast distribution of the mean for dry yield data in test and control groups of wheat fields is outlined in the provided Python code snippet (Listing 2). The process commences with the extraction of the necessary dry yield data, followed by the establishment of probabilistic models for both the test and control groups. Subsequent to the generation of samples from the posterior distributions of these groups, the computation of the contrast distribution of the mean is concluded.

F4 illustrates the reconstructed dry yield distributions for wheat fields MU21, comparing fields that utilized green manure (TF) to those that did not (CF). The central image represents the contrast distribution, alternatively referred to as the posterior distribution of the difference. On the right, the posterior contrast is displayed, signifying the likelihood of measurements in the TF surpassing those in the CF. Essentially, this illustrates the proportion of Test group measurements that exceed those of the CF.

The posterior distribution of the difference between the TF and CF groups serves as a robust metric, accurately quantifying the effectiveness of the treatment administered to the Test group in contrast to the CF. It is not permissible to simply compare the overlap in distributions. The contrast distribution indeed embodies this difference.

When evaluating the wheat fields MU22, the mean difference value between fields with and without wheat stands at 2860, a value significantly and reliably above zero. However, it is important to note that the overlapping distributions of the TF and CF groups do not denote a reliable difference. The contrast distribution essentially comprises the distribution of the difference between the simulated measurement pairs for the TF and CF groups. Furthermore, the overlap of distributions should not be misconstrued as an indication of identical distributions.

##### 2.3.1.3 Statistical considerations of the impact of the unobserved factors

It is important to note that the yield data we analyzed were not direct observations of the actual yield, but rather of yield monitor measurements. As such, there is a potential for error introduced by the yield monitor system. However, the precision of the yield monitor system is sufficiently high, minimizing the impact of this potential error impact on our analysis.

In addition to yield monitor error, we also considered the heterogeneity of the observed fields and other unobserved factors such as variations in humidity and other environmental conditions. These factors can significantly influence crop yields and, if not properly accounted for, could introduce bias into results.

To mitigate the impact of these unobserved factors, we adopted a comparative approach for each observation. Specifically, we compared neighboring fields, ensuring that any unobserved factors would have a similar impact on both the test and control sets. This approach allowed us to isolate the effect of green manure application on crop yield from the effects of these unobserved factors.

To further address field heterogeneity, we divided each field into smaller polygons (**Figure 3**, with each polygon represented by a single yield monitor measurement. This approach allowed us to compare yields at the polygon level rather than at the field level, effectively canceling out the impact of field heterogeneity on our results.

By adopting these strategies, we were able to conduct a more robust and accurate analysis of the impact of green manure on crop yields, accounting for potential sources of error and bias. This rigorous approach to data analysis underscores the validity of our findings and their implications for sustainable agricultural practices.

## 3 Results

**Figure 4:**
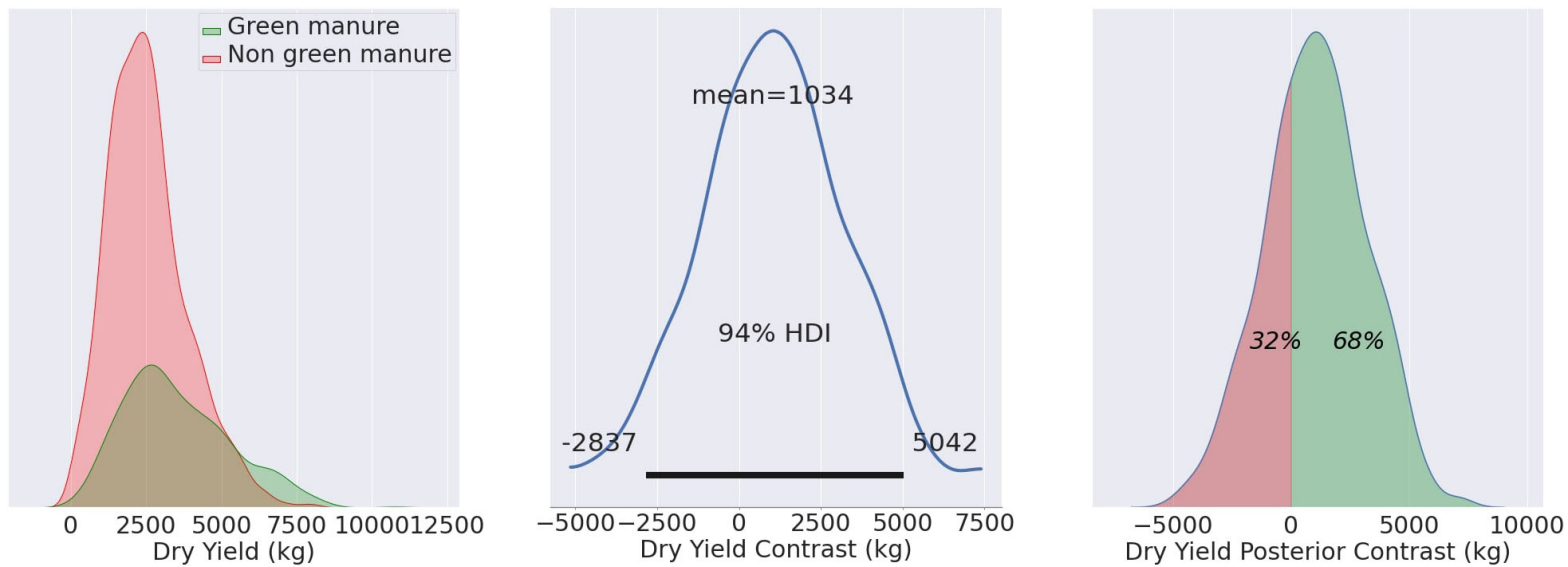
Mean yield values for MU21, showing the TF mean (3540 kg/ha), CF mean (2550 kg/ha), and contrast mean (1034 kg/ha). The effects are statistically evident in 68% of the total observed area.

**Figure 5:**
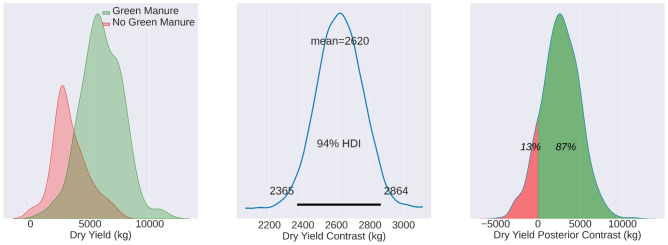
Mean yield values for MU22, illustrating the TF mean (5990 kg/ha), CF mean (3220 kg/ha), and contrast mean (2620 kg/ha). The effects are statistically evident in 87% of the total observed area.

**Figure 6:**
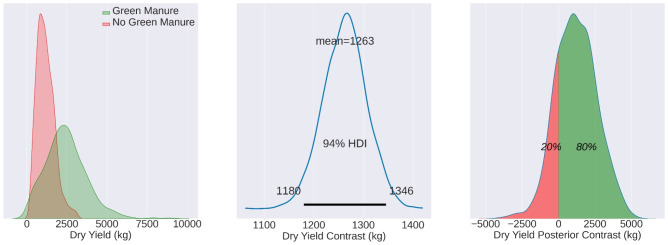
Mean yield values for DJ22, with the TF mean (2480 kg/ha), CF mean (1170 kg/ha), and contrast mean (1263 kg/ha). The effects are statistically evident in 80% of the total observed area.

The results of our methodology are summarized in **Table 6** and **Figures 4**–**6**, including mean yield values for both the TF and CF groups, as well as the contrast mean for MU21, MU22, and DJ22. In each case, the mean values are accompanied by the percentage of the total observed area where the effects are statistically evident, leaving the remaining percentage not statistically discernible due to other factors affecting yield.

**Table 6:** Experimental results for the effectiveness of green manure on crop yield.

Observation	Yield (kg/ha)			Gain (%)
	*µ*Test	*µ*Control	*µ*Contrast mean	
MU21	3540	2550	1034	39
MU22	5990	3220	2620	86
DJ22	2480	1170	1263	112

For the growing season of MU21, the application of green manure resulted in a TF yield average of 3540 kg/ha, compared to the CF average of 2550 kg/ha. The calculated contrast mean yield, standing at 1034 kg/ha, represents the mean of the differences between matched pairs of TF and CF, rather than the difference of the group means. This nuanced approach, which is discernible across 68% of the observed area, underpins the agronomic benefits of green manure, as evidenced in **Figure 4**.

Moving on to MU22, the mean yields for the TF and CF groups were 5990 kg/ha and 3220 kg/ha, respectively. The contrast mean was notably higher at 2620 kg/ha, with the effects being statistically evident in an impressive 87% of the observed area. This season’s results, depicted in **Figure 5**, further corroborate the efficacy of green manure in enhancing crop productivity.

Lastly, in DJ22, the mean yields for the TF and CF groups were 2480 kg/ha and 1170 kg/ha, respectively. The contrast mean here was 1263 kg/ha, and the effects were statistically discernible in 80% of the observed area, as illustrated in **Figure 6**.

The central panel in each of **Figures 4**–**6** represents the contrast distribution - a Bayesian estimation of the difference between yields in TF and CF, computed for each matched pair rather than derived from the simple difference in group means. This estimation accounts for the variability within each pair, providing a more accurate depiction of the effect size. The figures printed on the central panel indicate the HDI, which contains the range of most credible values for the contrast mean.

The right-most panels in these figures reflect the posterior distribution of the contrast estimates, with the percentages indicating the probability of the TF yields exceeding the CF yields. It’s noteworthy that these distributions appear non-normal, which may be attributed to the Bayesian estimation process that accounts for prior information and the data’s inherent variability, rather than assuming a normal distribution for the yield data.

**Figure 7:**
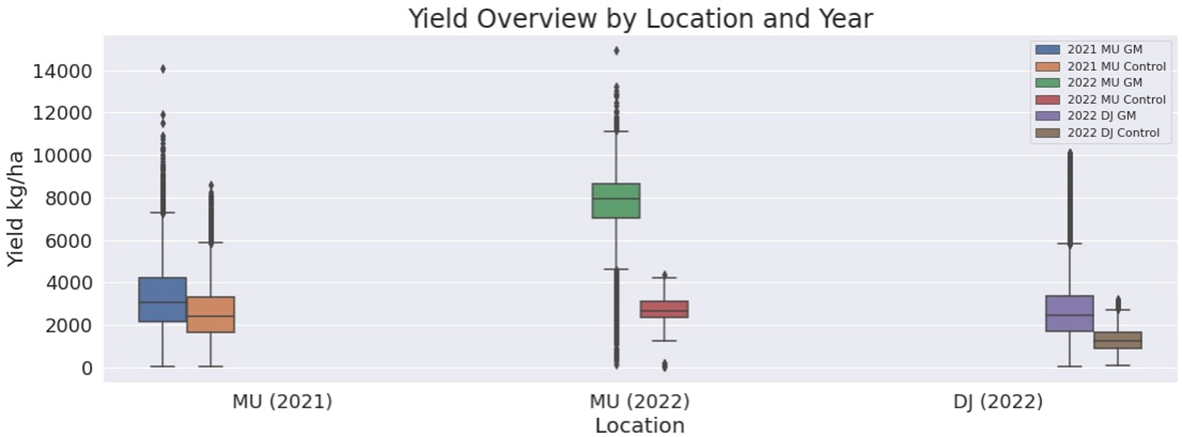
Box plots of the yield values for the test and control groups in MU21, MU22, and DJ22. the figure illustrates the differences in yield associated with the application of green manure (2021 MU GM, 2022 MU GM, and 2022 DJ GM) and control groups (2021 MU Control, 2022 MU Control, and 2022 DJ Control).

These comprehensive results, through rigorous statistical analysis, consistently reinforce the positive impact of green manure on crop yields across different locales and seasons. The visual comparison provided in **Figure 7** through box plots of the yield values for the TF and CF in MU21, MU22, and DJ22, conveys the substantial and consistent yield improvement associated with green manure application, underscoring its potential as a sustainable agronomic practice.

## 4 Discussion

In this research, we endeavored to elucidate the effects of green manure application on wheat yields over two consecutive growing seasons, integrating novel methods that move beyond traditional trial plots to encompass larger field scales. Our results affirm the benefits of green manure, echoing the findings of N’Dayegamiye and Tran (2001), who reported similar enhancements in wheat yields and nitrogen uptake.

Mineral nitrogen provision is a key factor in the response of cereals following legumes compared with cereals following non-legumes Peoples and Herridge (1990); Chalk (1998); Evans et al. (2001); Peoples et al. (2009). However, the response in wheat grain yield may not be entirely due to plant available N. Improvements in soil structure, phosphorus mobilization, the breaking of pest and disease cycles which afflict cereal monoculture, and phytotoxic and allelopathic effects of different crop residues have all been implicated in the yield response Ma et al. (2021). The yield benefits, which encompass a wide range of experimental results, vary from -0.2 to +3.1 Mg/ha extra yield (-11 to +156% of the reference yield) for temperate sites and from -2.1 to +3.0 Mg/ha extra yield (-44 to +265% of the reference yield) for Mediterranean sites Preissel et al. (2015). Peoples and Herridge (1990) cite studies by various authors that highlight the beneficial effects of warm-season legumes used as preceding crops on wheat grain yield. The observed increase in wheat grain yield, compared to a cereal-cereal cropping sequence, varied from 0.27 to 1.6 t/ha, with a relative increase ranging between 10% and 98%. In our research, as soil conditions degrade, the efficacy of green manure application becomes increasingly pronounced. Particularly in scenarios marked by poor soil quality, wherein the reference grain yield of wheat notably falls below the national average of 4,900 kg/ha Vucicevic (2023), the utilization of green manure demonstrates remarkable effectiveness. The amplification in wheat grain yield associated with the winter pea green manure application spanned from 1034 to 2620 kg/ha, and positive effects varied between 39% and 112%. Blanco-Canqui et al. (2012) reported that summer cover crops increased crop yields, particularly at low rates of N application. Without additional application of N mineral fertilizer, wheat yield was increased by 1.60 times. The incorporation of green manure crops into the soil at an optimal depth range of 20-25 cm likely indicates improved soil management practices, which in turn lead to enhanced soil structure, reduced compaction, and improved soil aeration Rinnofner et al. (2008); Tanveer et al. (2019). These favorable conditions foster the activity of microorganisms responsible for organic matter mineralization, ultimately resulting in increased mineral nitrogen provision Lyu et al. (2023). However, the novelty of our work lies not merely in reinforcing the advantageous outcomes of green manure but in the adoption of an innovative approach that evaluates these effects across expansive agricultural areas. The granularity of our field measurements, facilitated by dividing the fields into smaller polygons, allowed for a more detailed and accurate representation of each field, thereby overcoming the challenge of field heterogeneity—a factor often neglected in conventional small-scale trials. This methodological advancement has enabled us to detect yield variations with greater precision, as each polygon could be considered an independent experimental unit, providing a high-resolution dataset that captures the intricate interplays within a crop’s environment. Our use of Bayesian analysis and MCMC techniques represents a significant methodological leap in agricultural research. These techniques, as applied in the works of Besag and Higdon (1999) and Rodrigues-Motta and Forkman (2022), allow for a sophisticated interpretation of data that classical statistical methods may not fully capture. By integrating prior knowledge and considering the probability distributions of our data, we have unearthed a deeper understanding of the yield responses to green manure. It is crucial to note that yield monitor data, while invaluable, are not infallible proxies for actual yields. The potential errors inherent in yield monitor data were assumed to be minor and comparable across TF and CF, thus not substantially influencing the comparative analysis. Nevertheless, this assumption warrants further scrutiny, as any systematic discrepancies could affect the robustness of our conclusions. Our findings contribute to a growing body of evidence that supports the use of green manure as a sustainable agricultural practice, capable of boosting crop yields across diverse growing conditions. However, this study’s scope, confined to Mužlja and Ðurđevo, suggests the need for broader research across varying soil types, climates, and management practices to generalize these results. Future investigations should also delve into the long-term impacts of green manure on soil health and nutrient dynamics, which could offer insights into the sustainability of these practices. Moreover, advanced statistical models, such as dependency-extended two-part models Rodrigues-Motta and Forkman (2022), could refine our understanding of the intricate relationships within agricultural data. In conclusion, while acknowledging the limitations and assumptions inherent in our methodology, our research presents a compelling case for the adoption of yield monitor data in large-scale agricultural settings. By extending the application of rigorous, large-scale field analyses and advanced statistical techniques, future research can continue to unravel the complexities of sustainable farming practices, thereby enhancing the global agricultural landscape.

## 5 Conclusion

This study signifies a pivotal shift in agricultural research methodologies, integrating sophisticated statistical techniques such as Bayesian analysis and MCMC to evaluate green manure’s impact on large-scale fields over two growing seasons. These methodological advancements enhance the reliability of our findings, demonstrating the agronomic benefits of green manure through rigorous assessment of yield monitor data and field heterogeneity. Our results not only confirm the effectiveness of green manure in boosting crop yields but also highlight its role in promoting sustainable farming practices. The adoption of these advanced statistical methods provides a more solid foundation for decisions in sustainable agriculture, advocating for green manure’s integration into farming systems. Looking ahead, it’s crucial to explore green manure’s broader applications and its potential to improve soil health and agricultural sustainability. Our study presents a compelling case for the use of green manure as a scientifically backed strategy to enhance both yield and sustainability in agriculture, marking a significant advancement towards eco-friendly farming practices.

## Data availability statement

The raw data supporting the conclusions of this article will be made available by the authors, without undue reservation.

## Author contributions

NG: Data curation, Formal Analysis, Investigation, Methodology, Software, Visualization, Writing – original draft. ĐK: Methodology, Resources, Supervision, Validation, Writing – review & editing. MZ: Conceptualization, Investigation, Methodology, Validation, Writing – original draft, Writing – review & editing. ST: Writing – review & editing, Supervision.

## Appendix

**Listing 1.** Estimating *µ* and *σ*

**import** pymc3 as pm

**import** numpy as np

*# Assume that dry y i e l d measurements are stored in t h i s numpy array* dry_yield_measurements = np. array ([…]) *# replace with actual data*

*# Calculate prior mean and standard deviation from the measurements*

mu_prior = np. mean (dry_yield_measurements)

s t d _ p r i o r = np. std (dry_yield_measurements)

*# Create a PyMC3 model*

with pm. Model () as model :

*# Prior for mean and standard deviation*

mu = pm. Normal (‘mu’, mu=mu_prior, sd= s t d _ p r i o r)

sigma = pm. Exponential (‘ sigma ‘, 1/mu_prior)

*# Likelihood (sampling d i s t r i b u t i o n) of observations*

dry_yield = pm. Normal (‘ dry_yield ‘, mu=mu, sd=sigma, observed= dry_yield_measurements)

*# Perform MCMC sampling*

with model :

t r a c e = pm. sample (1000, tune =1000)

*# Convert the trace to a DataFrame for easier analysis and manipulation*

trace_df = pm. trace_to_dataframe (t r a c e)

**Listing 2.** Contrast Distribution Calculation

*# Extracting the dry y i e l d data from the t e s t and control groups*

d r y _ y i e l d _ t e s t = s e l f. g d f _ y i e l d _ t e s t [‘ DryYield ‘]. to_numpy ()

dry_yield_control = s e l f. gdf_yield_control [‘ DryYield ‘]. to_numpy ()

*# Calculating the prior mean and standard deviation from the grouped dry y i e l d data*

prior_mean = s e l f. gdf_dry_yield_grouped [‘ DryYield ‘]. mean ()

p r i o r _ s t d = s e l f. gdf_dry_yield_grouped [‘ DryYield ‘]. std ()

*# Defining the marginal l i k e l i h o o d s for the t e s t and control groups*

m a r g i n a l _ l i k e l i h o o d _ t e s t = 1

marginal_likelihood_control = 1

*# Constructing the p r o b a b i l i s t i c model for the t e s t group*

with pm. Model () as test_group_model :

mu = pm. Normal (‘mu’, mu=prior_mean, sd= p r i o r _ s t d)

sigma = pm. Exponential (‘ sigma ‘, 1/prior_mean)

dry_yield = pm. Normal (‘ dry_yield ‘, mu=mu, sd=sigma, observed= d r y _ y i e l d _ t e s t)

t r a c e _ t e s t = pm. sample (1000, tune =1000)

t r a c e _ d f _ t e s t = pm. trace_to_dataframe (t r a c e _ t e s t)

t r a c e _ d f _ t e s t [‘ c l a s s ‘] = s e l f. name_test_group

*# Constructing the p r o b a b i l i s t i c model for the control group*

with pm. Model () as control_group_model :

mu = pm. Normal (‘mu’, mu=prior_mean, sd= p r i o r _ s t d)

sigma = pm. Exponential (‘ sigma ‘, 1/prior_mean)

dry_yield = pm. Normal (‘ dry_yield ‘, mu=mu, sd=sigma, observed= dry_yield_control)

t r a c e _ c o n t r o l = pm. sample (1000, tune =1000)

t r a c e _ d f _ c o n t r o l = pm. trace_to_dataframe (t r a c e _ c o n t r o l)

t r a c e _ d f _ c o n t r o l [‘ c l a s s ‘] = s e l f. name_control_group

*# Concatenating the trace data frames for the t e s t and control groups* trace_df = pd. concat ([t r a c e _ d f _ t e s t, t r a c e _ d f _ c o n t r o l])

*# Generating samples for the control group using the p o s t e r i o r d i s t r i b u t i o n*

samples_control = np. concatenate (

[np. random. normal (m, s, 100) **for** m, s **in zip** (t r a c e _ d f _ c o n t r o l [‘mu ‘], t r a c e _ d f _ c o n t r o l [‘ sigma ‘])])

samples_control = np. random. choice (samples_control, size =1000, replace =True)

*# Generating samples for the t e s t group using the p o s t e r i o r d i s t r i b u t i o n*

samples_test = np. concatenate (

[np. random. normal (m, s, 100) **for** m, s **in zip** (t r a c e _ d f _ t e s t [‘mu’], t r a c e _ d f _ t e s t [‘ sigma ‘])])

samples_test = np. random. choice (samples_test, size =1000, replace =True)

*# Calculating the contrast d i s t r i b u t i o n of the mean* contrast_mean = t r a c e _ d f _ t e s t [‘mu’] − t r a c e _ d f _ c o n t r o l [‘mu’]

contrast_samples = samples_test − samples_control
